# GRID and Multiphonon States

**DOI:** 10.6028/jres.105.014

**Published:** 2000-02-01

**Authors:** S. J. Robinson

**Affiliations:** Department of Physics, Tennessee Technological University, Cookeville, Tennessee 38505

**Keywords:** deformed nuclei, multiphonon states, spherical nuclei

## Abstract

The development of the GRID technique for determining nuclear level lifetimes of excited low-spin states populated in thermal neutron capture reactions has resulted in the ability to perform detailed studies of proposed multiphonon excitations for the first time. This paper discusses the experimental evidence for multiphonon excitations determined using the GRID technique. In deformed nuclei several good examples of γγK^π^ = 4^+^ excitations have been established, whereas the experimental evidence gathered on K^π^= 0^+^ bands is contradictory, and any interpretations will likely involve the mixing of several different configurations. In vibrational nuclei the GRID technique has helped to establish the existence of multiple quadrupole phonon excitations in ^114^Cd, and an almost complete set of quadrupole-octupole coupled states in ^144^Nd.

## 1. Introduction

There is a long-recognized paradox in the interpretation of collective excitations in nuclei. On the one hand, purely collective models, such as the simple liquid drop model [1], treat the fundamental excitations as vibrations of the nuclear surface, or phonons. As such, these phonons are boson-like in character and multiple excitations of the same type are possible, resulting in high-lying “multiphonon” states. However, from a microscopic viewpoint, collective excitations are composed of a coherent superposition of quasiparticle excitations of fermions in orbits close to the Fermi surface. The Pauli principle then raises questions about the microscopic structure of states constructed from the coupling of like phonons, since only a limited number of particles can occupy each orbit. Indeed, there have been suggestions that, due to this effect, even two-phonon states in deformed nuclei would be highly fragmented [2]. From the collective viewpoint, the excitation energy of, and the absolute transition rates from, such multiphonon states can provide details of the structure of the phonons themselves, that the single-phonon excitations do not reveal.

Although of long-standing interest, the experimental study of such multiphonon states has proved to be difficult (especially in deformed nuclei) since they are generally of low spin, do not lie near to the yrast line (except for the J = 2*n* states in vibrational nuclei), and so are not populated easily in heavy-ion reactions. Although they are populated in the nonselective (n,γ) reaction, the inability to measure level lifetimes in the picosecond range meant that, in the absence of absolute transition rates, identification of such states had to be tentatively based on branching ratios. All this changed, however, with the development of the GRID technique [3] in the late 1980s. This development, together with experimental improvements in spectroscopy following Coulomb excitation [4] and inelastic neutron scattering [5], has made possible the measurement of lifetimes of excited low-spin states, and hence the extraction of absolute transition rates. This has, in turn, promoted more detailed studies of suggested multiphonon states and allowed us to answer some of these long-standing questions.

## 2. Deformed Nuclei

In deformed nuclei, vibrational excitations represent oscillations around the equilibrium deformed quadrupole shape. Because of the lack of spherical symmetry, quadrupole oscillations are of two types—usually called *β* and γ vibrations. While *β* vibrations represent oscillations in the degree of deformation, and as such have no projection of angular momentum on the symmetry axis (K^π^ = 0^+^), γ-vibrations are oscillations in which the nucleus takes on axially asymmetric shapes, and thus do have an angular momentum projection (K^π^= 2^+^). Two-phonon excitations can then be of four types, *ββ* (K^π^= 0^+^), *β*γ (K^π^= 2^+^), and γγ (K^π^= 0^+^ and K^π^= 4^+^). Of course, all of these excitations will have the usual rotational bands built upon them. Many examples of two-phonon bands have been proposed, but in all cases these assignments were based on E2 branching ratios, rather than absolute transition rates. Shortly after the development of the GRID technique, it was soon realized that it could be used to measure lifetimes, and hence extract absolute *B*(E2) values, for proposed two-phonon states, and so provide the first definitive evidence for (or against) the two-phonon interpretation for some of these states.

### 2.1 Gamma Vibrations

While the single γ vibration is a well established feature across a wide range of deformed even-even nuclei [1], the question of the existence of γγ vibrations has only recently been resolved. The single phonon γ excitation is characterized by a K^π^ = 2^+^ excited rotational band at an energy of around 1 MeV, the 2^+^ member of which decays to the 0^+^ ground state via a moderately collective E2 transition with a strength of several W.u. In the harmonic limit the γγ (K^π^ = 4^+^) vibration should lie at twice the energy of the single-phonon excitation (where two-quasiparticle excitations are also possible) and decay to it with a transition strength of 2.7 times the γ to ground transition. However, anharmonicities can change both the excitation energy and transition rate from the two-phonon state. Predictions from a wide range of models indicate that the γγ (K^π^= 4^+^) state should lie at about 2.5 times the energy of the single γ excitation and have an E2 transition to it of about the same strength as the γ to ground state transition.

In a landmark experiment [6], a GRID measurement of level lifetimes in ^168^Er provided the first direct evidence, from absolute transition rates, for the existence of an essentially intact γγ (K^π^= 4^+^) excitation. This study showed that an established K^π^= 4^+^ bandhead at 2.055 MeV had a collective E2 decay (of between 2.5 W.u. and 7.5 W.u.) to the established γ bandhead at 821 keV. Further, the ratio *B*(E2: 4^+^γγ → 2^+^γ)/*B*(E2: 2^+^γ→ 0^+^g) was determined to be in the range 0.5 → 1.6, in excellent agreement with several theoretical models. Two further experiments, using Coulomb excitation, have since confirmed this result, as shown in Table 1. Since this first study, another GRID experiment has provided good evidence for a γγ (K^π^= 4^+^) excitation in ^164^Dy [9], while other techniques have been used to identify such states in ^166^Er [10], ^192^Os [11], and ^232^Th [12]. Thus it seems that the γγ (K^π^= 4^+^) mode is well established across a wide range of deformed nuclei and the systematics of similar K^π^= 4^+^ bands seem to suggest it is a common feature [13]. Information on the γγ(K^π^= 0^+^) mode is less complete, as will be discussed in the next section.

### 2.2 K^π^ = 0^+^ Bands

Many examples of low lying K^π^= 0^+^ bands are known in deformed nuclei and the traditional interpretation of the lowest of these has been in terms of a *β* vibration. However, the behaviour of these excitations is far from systematic, with large fluctuations in both excitation energy and transition strengths, leading to several different theoretical interpretations [1,14,15].

Again, the GRID technique has been used to try and investigate the nature of these low lying K^π^= 0^+^ bands, but so far a consistent picture has not emerged. In particular, a GRID study of the 2^+^, 4^+^, and 6^+^ states in the lowest K^π^= 0^+^ band in ^168^Er [16] showed that these states decay more strongly to the γ band than to the ground band, and thus led to an interpretation of a large γγ (K^π^= 0^+^) component in this band. However, a Coulomb excitation study of the 0^+^ bandhead [8] concluded that there was no γγ component in this state. A similar contradictory picture has emerged recently in a GRID study of ^178^Hf [17], where different interpretations of the first excited K^π^= 0^+^ band emerge, depending on which band members one looks at. Also in this nucleus it has been shown that the $0_{4}^{+}$ band decays strongly to the first excited K^π^= 0^+^ band, leading to a possible *ββ* interpretation [17]. Meanwhile, a GRID experiment on ^164^Dy [16] has suggested that the lowest excited K^π^= 0^+^ band in this nucleus must be of quasiparticle nature, with both the *β* and γγ (K^π^= 0^+^) lying at higher energies. This, at least, is in agreement with a study of ^166^Er [10], which concluded that the $0_{4}^{+}$ and $0_{5}^{+}$ bands represented the best candidates for the *β* and γγ (K^π^= 0^+^) vibrations.

It thus seems that there is not yet a consistent picture regarding the interpretation of low lying K^π^= 0^+^ bands in deformed nuclei. However, it seems likely that, given the contradictory evidence so far, a mixing of all possible configurations will be needed, and much more experimental evidence will be required to test model predictions of this sort.

## 3. Vibrational Nuclei

In spherical nuclei, the absence of a definite symmetry axis means that all quadrupole vibrational modes are degenerate, and so all quadrupole multiphonon states must be built using only one type of phonon, with no rotational bands built on them. This leads to the well-known vibrational spectrum with a two-phonon triplet, three-phonon quintuplet, etc. Many spherical nuclei also exhibit a low-lying 3^−^ state with a collective E3 transition to the ground state. This can be explained using an octupole phonon in which the nuclear surface oscillates asymmetrically about its spherical equilibrium shape. Thus, in vibrational nuclei, excited collective states can be constructed from multiple excitations of both the elementary quadrupole (2+) and octupole (3^−^) phonons.

### 3.1 Multiple Quadrupole Excitations

Many reasonable examples exist of proposed two-phonon quadrupole excitations with established collective transitions to the one-phonon 2^+^ state. There are also examples of states identified in heavy ion reactions which seem to correspond to the J = 2*n* member of multiplets with up to six quadrupole phonons [18,19]. However, examples of complete n-phonon multiplets, in which both excitation energies and E2 transition rates are close to those predicted in the harmonic limit, are much rarer [20]. This paucity of good examples is, presumably, due to the fact that higher-n multiplets lie at energies near, or above, the pairing energy and so can mix strongly with two-quasiparticle configurations. In addition collective particle-hole excitations across shell closures can lead to low-lying intruder states which may mix with the normal states. This situation was examined in detail in a GRID measurement of lifetimes in ^114^Cd [21], where it was shown that the experimentally determined E2 transition rates could be explained, either in terms of no mixing between normal and intruder configurations, or with a particular mixing in which there is an accidental double cancellation. Either way, it does seem that some cohesive n-phonon structures do persist at higher excitation energies, and the even-even Cd nuclei provide a good testing ground for studying this phenomenon.

### 3.2 Multiple Octupole Excitations

Though many good examples of single collective octupole vibrations exist in even-even nuclei near closed shells, little is known experimentally about two-phonon octupole states. In the harmonic limit this coupling produces a quartet of states (0^+^,2^+^,4^+^, and 6^+^) at twice the single phonon energy, which decay via collective E3 transitions to the single phonon state. However, due to the difficulty of measuring E3 transitions, E1 transition strengths have been used as evidence of two-phonon octupole character [22]. The two best prospects for the definite identification of two-phonon octupole states seem to be ^146^Gd [23] and ^208^Pb [24]. Unfortunately the GRID technique is not likely to be useful in this case, because the expected lifetimes of two-phonon octupole states are in the range of tens to hundreds of picoseconds, and thus too long to be measured by GRID.

### 3.3 Quadrupole-Octupole Coupled Excitations

When coupling single quadrupole and octupole phonons, very different quasiparticle combinations are involved in the collective excitations. Because of this, the Pauli principle does not block the creation of such quadrupole-octupole coupled (QOC) states and so these should provide a clearer test of the coupling of collective modes. This coupling produces a quintuplet of negative parity states (1^−^ to 5^−^), which, in the limit of weak coupling, should lie at an energy which is the sum of the single phonon energies. Further, the E2 and E3 transitions from these states to the $3_{1}^{-}$ and $2_{1}^{+}$ states should be of the same strength as the $2_{1}^{+}\to 0_{1}^{+}$ and $3_{1}^{-}\to 0_{1}^{+}$ transitions (which are typically tens of W.u.). Such E2 transition rates imply lifetimes which are measurable by GRID and, indeed, a GRID experiment did provide strong evidence that a group of negative parity states at around 2 meV to 3 MeV in ^144^Nd provided a good example of QOC states [25]. However, the large errors on the measured E2 and E3 transition strengths could not rule out substantial mixing with quasiparticle states and subsequent splitting of the QOC strength. Also, the transition from the proposed QOC 4^−^ state at 2205 keV to the $3_{1}^{-}$ level had a measured mixing ratio which indicated essentially no E2 component in this transition [26].

Since then further studies have been carried out to reduce the errors on these transition strengths, and the results of these firmly establish a set of QOC states in this nucleus. Firstly, the branching ratio of the very weak 1397 keV (5^−^→ 2^+^) transition was measured [27]. More recently, further GRID lifetime measurements of the proposed 4^−^, 5^−^, and 3^−^ QOC states have been made, and the M1/E2 mixing ratio of the $4_{\mathrm{QOC}}^{-}\;\to \;3_{1}^{-}$ and the $3_{2}^{-}\to 3_{1}^{-}$ transitions have been determined [28].

Table 2 shows the absolute E2 and E3 transition rates (in W.u.) for the relevant transitions in ^144^Nd, determined using the current data. Evidently the E2 transitions strengths from the $1_{1}^{-}$, $5_{1}^{-}$, and $4_{1}^{-}$ states to the $3_{1}^{-}$ state are all the same size as the $2_{1}^{+}\to 0_{1}^{+}$ transition, and thus are a strong indication of the near-harmonic QOC nature of these states. In addition, this interpretation is supported for the $5_{1}^{-}$ state by the strength of the E3 transition to the $2_{1}^{+}$ state. The E2 strength of the $3_{2}^{-}\to 3_{1}^{-}$ transition is also enhanced, though not to the same degree. However, its strength is consistent with that predicted by Vogel and Kochbach [32] for the $3_{\mathrm{QOC}}^{-}$ state and it is adopted as such. From this evidence, it is clear that ^144^Nd provides the clearest example to date of QOC states. With this interpretation only the 2^−^ member of the QOC quintuplet is missing, and there is good reason to believe that this state lies at an energy of above 3 MeV [25].

## 4. Conclusion

Since the development of the GRID technique it has played a leading role in the study of multiphonon states of all types. Though there are still many open questions in this field, especially concerning deformed Kπ = 0^+^ bands, it is now becoming clear that cohesive multiphonon excitations persist to higher excitation energies than previously suspected [33] and GRID measurements have provided significant evidence for this pervasive phonon structure of nuclear excitations.

## Figures and Tables

**Table 1:** Experimental determinations of the ratio: *R*(4^+^) = *B*(E2: 4^+^γγ → 2^+^γ)/*B*(E2: 2^+^γ → 0^+^g) for ^168^Er

Technique	*R*(4^+^)	Reference
GRID (n, γ)	0.5 → 1.6	[6]
Coul. ex.	1.4 ± 0.4	[7]
Coul. ex.	2.2 ± 0.5	[8]

**Table 2:** Absolute rates (in W.u.) for E2 and E3 transitions from negative parity states in ^144^Nd

Initial state	Final state	Transition energy (keV)	Multipolarity	*B*(EL)^a^ (W.u.)
$2_{1}^{+}$	$0_{1}^{+}$	696.5	E2	24.4(3)^b^
$3_{1}^{-}$	$0_{1}^{+}$	1510.7	E3	33.9(17)^c^
$5_{1}^{-}$	$2_{1}^{+}$	1396.6	E3	32(9)
	$3_{1}^{-}$	582.5	E2	25(6)
$1_{1}^{-}$	$3_{1}^{-}$	675.1	E2	20(5)^d^
$4_{1}^{-}$	$3_{1}^{-}$	693.9	E2	$30{(}_{-9}^{+12})$
$3_{2}^{-}$	$3_{1}^{-}$	1095.2	E2	12(4)
	$0_{1}^{+}$	2605.8	E3	1.1(1)^e^

a Quoted errors include contributions from errors in level lifetimes, transitions branching ratios, and mixing ratios (where appropriate).

b Ref. [29].

c Ref. [30].

d Ref. [25].

e Ref. [31].
